# Machine Learning for Sarcopenia Prediction in the Elderly Using Socioeconomic, Infrastructure, and Quality-of-Life Data

**DOI:** 10.3390/healthcare11212881

**Published:** 2023-11-01

**Authors:** Minje Seok, Wooseong Kim, Jiyoun Kim

**Affiliations:** 1 Computer Engineering Department, Gachon University, Seongnam 13120, Republic of Korea; smj990203@gachon.ac.kr; 2 Convergence Health Science, Gachon University, Incheon 21936, Republic of Korea; eve14jiyoun@gachon.ac.kr

**Keywords:** sarcopenia, machine learning, explainable AI, socioeconomic, infrastructure, quality of life

## Abstract

Since the WHO’s 2021 aging redefinition emphasizes “healthy aging” by focusing on the elderly’s ability to perform daily activities, sarcopenia, which is defined as the loss of skeletal muscle mass, is now becoming a critical health concern, especially in South Korea with a rapidly aging population. Therefore, we develop a prediction model for sarcopenia by using machine learning (ML) techniques based on the Korea National Health and Nutrition Examination Survey (KNHANES) data 2008–2011, in which we focus on the role of socioeconomic status (SES), social infrastructure, and quality of life (QoL) in the prevalence of sarcopenia. We successfully identify sarcopenia with approximately 80% accuracy by using random forest (RF) and LightGBM (LGB), CatBoost (CAT), and a deep neural network (DNN). For prediction reliability, we achieve area under curve (AUC) values of 0.831, 0.868, and 0.773 for both genders, males, and females, respectively. Especially when using only male data, all the models consistently exhibit better performance overall. Furthermore, using the SHapley Additive exPlanations (SHAP) analysis, we find several common key features, which mainly contribute to model building. These include SES features, such as monthly household income, housing type, marriage status, and social infrastructure accessibility. Furthermore, the causal relationships of household income, per capita neighborhood sports facility area, and life satisfaction are analyzed to establish an effective prediction model for sarcopenia management in an aging population.

## 1. Introduction

In 2021, the World Health Organization (WHO) redefined the definition of aging from a functional perspective. This movement reflects the recent trend that the ability of the elderly to perform daily activities is crucial for quality of life (QoL), regardless of the presence of diseases. In other words, “healthy aging” can be defined as the ability of the elderly to still engage in daily activities even with the presence of diseases. Sarcopenia, defined as the substantial decline in skeletal muscle mass, can disrupt daily activities and significantly degrade the QoL of the elderly. According to [1], about 13% of 50 million sarcopenic individuals are between 60 and 70 years old. Therefore, the projected doubling of the global population over 60 years old by 2050 [2,3] is now becoming a great threat to public health.

Recently, South Korea has been considered one of the fast-aging countries in the world, in which the elderly population is expected to increase from 5.3 million (15.7% of the whole population) in 2022 to 19 million (40% of the whole population) in 2050 [4,5]. The Korean government recently assigned a code to sarcopenia in the Korean Standard Classification of Diseases 8th revision (KCD8) [6]. As a result, sarcopenia has gained attention as a prominent health concern in the elderly.

For this, the Korea National Health and Nutrition Examination Survey (KNHANES), spanning from 2008 to 2011, has been involved in numerous studies exploring the correlation between sarcopenia and various factors within the Korean elderly population. For instance, notable investigations, such as those presented in [7,8], examined the relationship between sarcopenia and cardiovascular disease. The connection between sarcopenia and pulmonary function was explored in [9,10,11,12]. In addition, Refs. [13,14,15] addressed the relationship between sarcopenic obesity and metabolic syndrome. Further research [16] unveiled that both elevated levels of physical activity (PA) and a higher ratio of energy intake to basal metabolic rate were independently linked to a reduced risk of sarcopenia [16]. Certain lifestyle activities, such as prolonged sleep duration [17] and regular walking [18], were also shown to reduce the risk for sarcopenia.

Various factors, such as a physical disorder, poor nutrition, inflammatory pathway activation, neurological decline, hormonal changes, fatty infiltration, and chronic illness, can induce sarcopenia as a multifactorial disease [19]. Low-level PA from sarcopenia can lead to secondary conditions, such as osteoporosis, diabetes, and cardiovascular diseases, as well as cognitive impairment. This cycle of decline has far-reaching effects on the overall body, causing social isolation, stress, depression, accelerated muscle loss, and ultimately mortality. Therefore, numerous screening methods based on those medial factors have been proposed in [20,21,22,23], along with international consensus efforts [1,24,25,26].

To promote “healthy aging”, it is essential to analyze not only the aforementioned physical, nutritional, and medical aspects but also consider it from a sociological perspective. Socioeconomic status (SES), in this regard, has long been associated with health and encompasses factors, such as income, education, and occupation. Several studies have introduced the relationship between SES and sarcopenia. Dorosty et al. [27] investigated the prevalence of sarcopenia and its association with SES among the elderly in Tehran. The results showed a decreasing prevalence of sarcopenia based on income levels, i.e., 20.5%, 18.2%, and 12.8% for low-, middle-, and high-income levels, respectively. Education level also affects the prevalence of sarcopenia, with 18.6%, 16.2%, and 12.3% prevalence for illiterate, primary school, and diploma levels, respectively. In a dataset from Wave 1 of the Irish Longitudinal Study on Ageing 2009–2011, Swan et al. [28] found that among a total 3342 community-dwelling older adults, 23.4% were classified as sarcopenic. Notably, there was an unequal distribution with prevalence rates of 28.9% among those with low educational levels and 18.1% among those with high educational levels. Moreno-Franco et al. [29] showed the relationship between SES and frailty by using the data of the Seniors-ENRICA study in Spain in 2012. Gandham et al. [30] found that the elderly with a tertiary degree of education and older adults living in advantaged areas were significantly less likely to suffer from obesity than others according to an analysis of the data from the Tasmanian Older Adult Cohort study. Based on such a relation, we utilize the SES of the KNHANES dataset to establish a sarcopenia prediction model.

Because sarcopenia can reduce QoL gradually without significant symptoms in its early stages, it is important to measure the physical and psychological health state through a QoL survey. For instance, Go et al. [31] found that sarcopenia is linked to reduced bone mineral density in Korean men, and it negatively affects their quality of life, especially in terms of mobility and usual activities. Furthermore, Tsekoura et al. [32], through an extensive literature review, demonstrated that individuals with sarcopenia have a significantly higher prevalence of issues related to various dimensions of QoL. Therefore, we adopt a general QoL survey, EuroQol (EQ-5D-3L) [33], to measure the daily health condition associated with sarcopenia, while it would be more insightful to utilize a disease-specific QoL questionnaire, the SarQoL, for sarcopenic subjects.

Furthermore, social infrastructure factors can have both direct and indirect impacts on health. Davern et al. [34] argued that social infrastructure, as a lifelong social service, contributes to promoting health and well-being; thus, the poor planning of social infrastructure has been linked to area-based health inequities. In the Korean Frailty and Aging Cohort Study, Seo et al. [35] showed that neighborhood environmental characteristics, such as the absence of public transportation, poor recreational facilities access, and a lack of traffic safety, are linked with an increased likelihood of sarcopenia. Meanwhile, the neighborhood environmental characteristics had limited effects on the change in skeletal muscle mass and grip strength for older adults living in rural areas [36]. Consequently, the effect of public infrastructure on the prevalence of sarcopenia can vary according to its type and location. In this study, we use the number and size information of only cultural facilities, social welfare facilities, senior recreation centers, and parks.

Although there have been numerous studies to build a sarcopenia prediction model using machine learning, most of them have primarily focused on training with medical or physiological factors. This is the first study, to the best of our knowledge, that utilizes sociological factors such as SES and social infrastructure in addition to QoL related to health state. For comparison, we conduct experiments with various feature combinations. In this study, we consider random forest (RF), LightGBM (LGB), CatBoost (CAT), and deep neural network (DNN) algorithms; LGB and CAT are based on gradient boosting trees, RF is based on an ensemble of multiple decision trees, and the DNN is based on multiple hidden neural layers. As a result, we successfully identify sarcopenia with approximately 80% accuracy by using various ML technique. Moreover, despite class imbalance, AUC values of 0.831, 0.868, and 0.773 are achieved for both genders, males, and females, respectively, which is the reliability performance of the binary classification. CAT is the model that provided the highest results across a variety of feature combinations and genders, highlighting its robustness. Especially when using only male data, all the models consistently exhibit better performance overall. We also apply oversampling, but there is no significant difference in the results.

To identify the contributing factors in predicting sarcopenia, we analyze the features considering the following two perspectives. The first aims to identify features with a significant influence on the predictions in the models. For this, the SHapley Additive exPlanations (SHAP) [37] that are based on the Shapley values [38,39] derived from cooperative game theory are used, which allows for quantifying the contribution of each feature to the model’s output, providing a numerical representation of how much each feature contributes to the predictions. Additionally, we score the top influential features in each model, identifying universally effective features for learning. The result shows that features such as monthly average household total income, housing type, marriage status, and the accessibility of social welfare facilities and senior leisure welfare facilities play an important role for learning across the aforementioned models in addition to basic features, such as age, BMI, and waist circumference. These common important features can help feature selection to improve model performance in the future.

The second perspective seeks to identify causal features that increase the risk of sarcopenia, helping to understand the fundamental nature of sarcopenia. The causal relationships between the specific feature and sarcopenia are investigated by causal inference techniques, allowing for the precise understanding of the impact of specific features on sarcopenia. Because sarcopenia is associated with age, age is set as a conditioning variable along with various confounding variables, and the conditional average treatment effect (CATE) is calculated. From the treatment effect (TE), we can gain insights into how sarcopenia responds to treatment in the context of varying ages. We have observed that a high household income quartile for households, life satisfaction, and body mass index (BMI) all play a positive role in reducing the risk of sarcopenia, and this effect becomes stronger with age. Additionally, the per capita area of neighborhood sports facilities positively contributes to the risk reduction; however, its impact diminishes with age. Understanding these causal relationships can help develop more accurate strategies for the prevention and management of sarcopenia.

The remainder of this paper is structured as follows: In the next section, we introduce previous studies about machine learning for sarcopenia prediction. In the Materials and Methods, we describe the methods for measuring muscle mass and diagnosing sarcopenia based on cutoff values and the dataset used in this study. We explain the features related to SES, social infrastructure, and QoL used in ML and present the statistical analysis results of these features. We then subsequently introduce ML techniques and analyzing methods, such as SHAP and causal inference. We present the experimental results of the models with diverse metrics and provide additional discussions on feature importance and causality in the following section. Finally, we conclude this paper with the limitations of this study and possible future works.

## 2. Machine Learning for Sarcopenia Prediction

Machine learning (ML) techniques, widely employed in numerous classification and prediction problems across various domains, have demonstrated their effectiveness and reliable performance by learning from diverse datasets. There have been many studies exploring machine learning techniques to establish sarcopenia prediction models using various features. Our previous survey on machine learning application for sarcopenia describes the details of machine learning algorithms and their pros and cons for the sarcopenia prediction model [40]. In this section, we introduce several studies on sarcopenic machine learning according to the selected features.

Several studies explore key features across all possible criteria and train the machine with them. For example, Kaur et al. [41] developed the logistic regression model using obesity-related features such as PA, protein intake, fat, diabetes, and body composition, achieving almost 97.69% accuracy in predicting outcomes as it is known that the obesity-related features contribute highly to prediction accuracy. Kang et al. [42] extracted features of intake information and nutrition status by using random forest and achieved an AUC value of about 0.8 across the linear regression (LR), gradient boost (GB), and support vector machine (SVM) algorithms. Luo et al. [43] also predicted sarcopenia by using features of electronic health records that include all basic health, clinical, and biological information. In these cross-sectional studies, feature selection algorithms result in different outcomes in terms of features and accuracy. Therefore, efforts to develop feature selection algorithms for sarcopenia have been made recently [44].

Several studies focus on PA status features as they are tightly related to sarcopenia. Ko et al. [45] achieved a 95% sarcopenia prediction accuracy of the female elderly by an SVM-based kinematic data analysis on an inertial measurement during walking. Kim [46] demonstrated the effectiveness of predicting sarcopenia only with physical characteristics and activity information that achieved over 80% accuracy by using an SVM, highlighting its competitiveness with other algorithms. However, SVM limitations for sarcopenia prediction include dependency on extensive datasets, sensitivity to feature selection, and concerns about generalizability across diverse populations. Previously, we also established sarcopenia prediction models by using the LR, RF, SVM, LGB, and DNN algorithms with the available PA data [47], as the relationship between low-level PA and risk for sarcopenia is well known in [48,49,50,51,52]. Our model showed a prediction accuracy of 84% and an AUC value of 0.88 by using a DNN with PA features. In particular, LGB and RF showed strong performance, with AUC values exceeding 0.9. The prediction performance of our model was notable compared to other approaches. Accordingly, we gained insights into the effective sarcopenia detection capabilities of ML and the vulnerability associated with specific factors.

Clinical features can be also used for machine training because the correlation of some of them to the sarcopenia has been revealed in previous studies. For instance, Tseong et al. [53] investigated the different diagnostic performance of different machine learning models in identifying sarcopenia in Crohn’s disease. Kim et al. [54] obtained an AUC value of approximately 0.74 by using ophthalmological examinations and demographic data. Liao et al. [55] used machine learning and medical data such as blood glucose, parathyroid hormone, total bilirubin, post-dialysis creatinine, etc., to develop two simple sarcopenia identifications for maintenance hemodialysis (MHD) patients. For patients undergoing peritoneal dialysis (PD), Wu et al. [56] used 12 core features of them for SVM and DNN models, achieving an AUC of 0.82 (95% confidence interval: 0.67–1.00).

Recently, there have been attempts to use X-ray, CT, and MRI images of patients for sarcopenia diagnosis and prediction because object detection and classification in images is one of the most developed techniques in machine learning. Burns et al. [57] estimated sarcopenia by abdominal CT images at multiple lumbar vertebral levels. Dong et al. [58] identified sarcopenia of advanced non-small-cell lung cancer (NSCLC) patients by using LightGBM with chest CT radiomics instead of the abdominal CT images. According to hyperparameter tuning, they could achieve 94% accuracy. Kim et al. [59] extracted the radiomic features from L3 CT images of the entire muscle area and partial areas of the erector spinae collected from non-small-cell lung carcinoma (NSCLC) patients and trained several models. The results showed the highest AUC as 0.837 from XGB. Ryu et al. [60] used a chest X-ray image for sarcopenia diagnosis and showed AUC results of 0.81 and 0.78 for internal and external test sets, respectively. Image learning is effective, but prediction accuracy can vary with image quality and the body part. Based on this trend, fully automated diagnosis based on the medical image is now under standardization [61].

Many recent studies demonstrate the feasibility of the machine learning technique for sarcopenia diagnosis and prediction by using various factors. However, it is a new approach in this study to predict sarcopenia by using the sociological data of the elderly in addition to the minimum health information.

## 3. Materials and Methods

### 3.1. Muscle Mass Measurement Techniques

Diagnosing sarcopenia involves assessing measurable factors, such as muscle mass, muscle strength, and physical performance, with muscle mass as an objective indicator and least affected by measurement environment and circumstance.

In diagnosing sarcopenia, the European Working Group on Sarcopenia in Older People (EWGSOP) has published guidelines regarding suitable measurement techniques for diagnostic parameters, such as muscle mass. Body imaging methods and bioelectrical impedance analysis (BIA) are recommended to evaluate muscle mass [24].

Several imaging techniques, such as computed tomography (CT), magnetic resonance imaging (MRI), and dual-energy X-ray absorptiometry (DXA), are available for estimating muscle mass or lean body mass. CT and MRI provide highly precise muscle mass values; however, there are concerns related to their elevated costs and radiation exposure. Consequently, DXA is preferred as a research-oriented measurement method due to its lower radiation exposure and comparable accuracy. Hence, we consider the DXA measurement result for the diagnosis of sarcopenia in this study.

BIA is a cost-effective approach for estimating fat mass and lean mass, by which diagnosis results exhibit a strong correlation with MRI-based prediction [62,63]. Moreover, it can be applied not only to ambulatory patients but also to those confined to beds. Accordingly, BIA appears as a promising alternative to DXA for assessing sarcopenia, although many of the recent portable devices provide only an approximated measurement for the entire body.

### 3.2. Sarcopenia Diagnosis and Cutoff Values

In this section, we provide the criteria for diagnosing sarcopenia based on muscle mass, including the specific measurement method and its corresponding cutoff value.

Initially, Baumgartner et al. [64] defined the diagnostic criteria of sarcopenia for the DXA method in 1998, relying on the appendicular skeletal muscle mass (ASM) and excluding the bone mass and fat mass in the extremities. The ASM was adjusted by height (ht) for its increment against height, as ASM/ht${}^{2}$ (kg/m${}^{2}$). The cutoff values were determined by 2σ (standard deviation) below the mean values for young adults aged 18–40 years, i.e., 7.26 and 5.45 for men and women, respectively, in the Rosetta Study reference data.

In 2003, Newman et al. [65] proposed two approaches for the diagnosis of sarcopenia by using DXA. In the first approach, the cutoff values were decided by the 20%-ile of the distribution for their gender instead of the previous cutoff based on the younger population. The induced cutoff values were comparable by 7.23 kg/ht${}^{2}$ and 5.67 kg/ht${}^{2}$ for men and women, respectively, with the previous suggestion. In the second approach, from a linear regression analysis to investigating the association between appendicular lean mass (aLM) with both height and fat mass, the cutoff value was derived by the 20%-ile of the distribution of residuals. Accordingly, the second approach achieved being able to identify 11.5% and 21.0% of men and women, respectively, in the obese group as sarcopenic, while the first approach did not detect sarcopenic cases among obese individuals.

From the standardization, the EWGSOP provided an operational definition and diagnostic strategy for diagnosing sarcopenia in 2010 [24]. In addition, the Asian Working Group for Sarcopenia (AWGS) developed cutoff values suitable for Asians [25] in 2014.

In this study, we use the cutoff values based on the appendicular skeletal muscle mass index (ASMI) measured by DXA rather than other muscle strength and physical performance. For this, the AWGS suggests using −2σ of the mean muscle mass for a young reference group or using the lower 20%-ile of the population. Therefore, the cutoff values are 7.0 kg/m${}^{2}$ and 5.4 kg/m${}^{2}$ in men and women, respectively, according to height-adjusted ASM defined by ASM/height${}^{2}$ (kg/m${}^{2}$) that the AWGS recommends instead of weight-adjusted ASM.

### 3.3. Participants and Data Selection

This research is founded on the KNHANES 2008–2011 by the Korea Centers for Disease Control and Prevention, operating under the Ministry of Health and Welfare. The KNHANES has three methods, namely, health surveys, nutritional assessments, and screening examinations, to catch the public health trends of the Korean population.

The health survey encompasses a broad spectrum of inquiries, including SES and QoL indicators, such as education, economic engagements, and overall well-being, as interest areas for this study. The nutritional assessment focuses on dietary patterns, nutritional knowledge, and the frequency of food consumption, among others. The screening examination involves measuring physical parameters, such as blood pressure, heart rate, and chest X-rays, among other metrics. In screening examinations, the body fat assessments derive bone mineral content and lean mass, which are critical factors for diagnosing sarcopenia. The appendicular skeletal muscle mass can be calculated by using these measurements acquired through DXA, which utilizes the differential transmission of radiation, with low-energy and high-energy X-rays penetrating soft tissues and bone tissues, respectively. It is important to note that this skeletal muscle mass assessment was exclusively conducted from 2008 to 2011, thereby restricting the dataset to that time frame.

In addition to the SES and QoL factors, we use information regarding accessibility and per capita area to social infrastructure facilities for training sarcopenia prediction models. We collect data related to the social infrastructure of 2008–2011 from the Korean Statistical Information Service (KOSIS) and several central administrative agencies. The Ministry of Health and Welfare (MOHW) provides the number of senior leisure welfare facilities [66]. The Ministry of Culture, Sports and Tourism (MCST) provides the number of cultural facilities [67] and the area of neighborhood sports facilities [68]. The area of urban parks [69] is sourced from the urban planning status statistic data provided by the Ministry of Land, Infrastructure and Transport (MOLIT). Additionally, the number of social welfare facilities [70] is aggregated from the statistical yearbooks for each administrative area. These five features are calculated for the following 16 metropolitan cities and provinces, with each region being distinguished by a city code: Seoul (1), Busan (2), Daegu (3), Incheon (4), Gwangju (5), Daejeon (6), Ulsan (7), Gyeonggi-do (8), Gangwon-do (9), Chungcheongbuk-do (10), Chungcheongnam-do (11), Jeollabuk-do (12), Jeollanam-do (13), Gyeongsangbuk-do (14), Gyeongsangnam-do (15), and Jeju-do (16). These infrastructure-related features are incorporated according to the residential regions of the participants.

**Figure 1:**
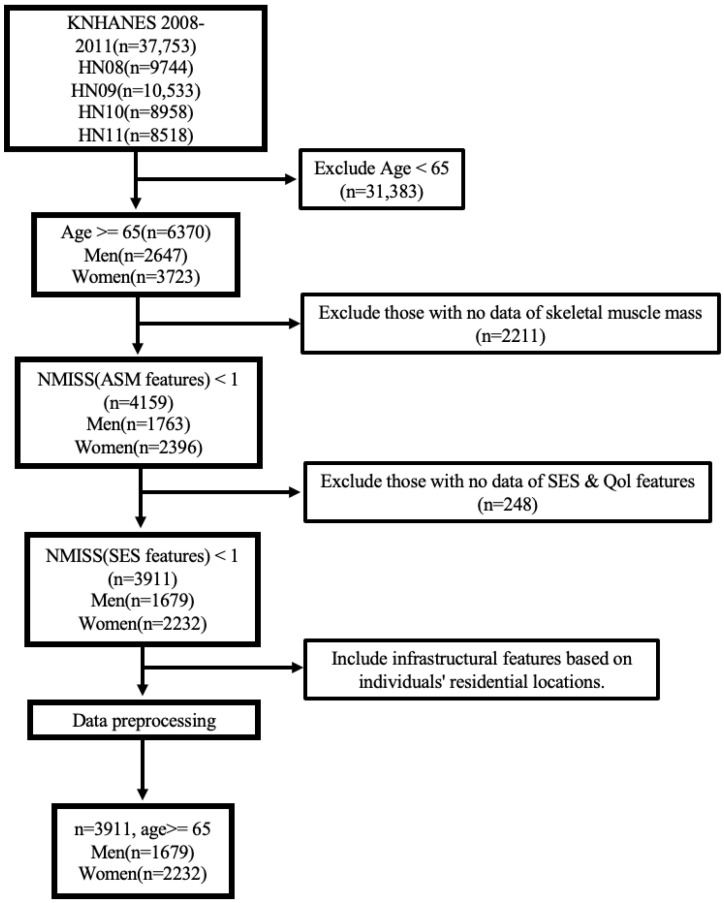
Process of subject selection, Korea National Health and Nutrition Examination Survey (KNHANES) IV and V (2008–2011).

Figure 1 illustrates the entire process of selecting the participants among the total 37,753 participants of the KNHANES. First, we exclude samples (*n* = 31,383) below the threshold of 65 years, following the criterion for defining senior citizens in the AWGS guidelines [25]. Second, we exclude individuals (*n* = 2211) without available skeletal muscle information enabling sarcopenia diagnosis. We then omit samples (*n* = 248) that lack SES and QoL features. Finally, we end up with a total of 3911 samples.

## 4. Feature Selection and Analysis

In this section, we introduce all the features regarding the SES, social infrastructure, and QoL features used for training the ML models available in the KNHANES and describe each individual feature and its preprocessing method if necessary. Additionally, we present a statistical analysis of the features in relation to sarcopenia.

### 4.1. Features of Socioeconomic Status

SES, including education, income, occupation, and housing, acts as a crucial metric of an individual’s or a household’s socioeconomic standing. It influences various factors related to a vulnerability to sarcopenia, nutritional status, living environment, and healthcare accessibility. According to [27,28,29,30], SES plays a pivotal role in identifying population groups at higher risk for sarcopenia.

First, economic activity status (EC1_1), basic livelihood allowance status (allownc), homeownership status (house), household composition code (genertn), marital status (marri_1), and marriage status (marri_2) are binary features, 0 or 1. The variables EC1_1, allownc, and house are encoded as 1 if yes and 0 otherwise. genertn is encoded as 1 for only sole-member households without a spouse or other family members and 0 otherwise. Marital status indicates the presence or absence of marriage, while marriage status refers to the current cohabitation status with a spouse. Married individuals are coded as 1 for marri_1. Especially, households currently cohabiting with spouses are encoded as 1 for marri_2. Second, the average monthly household income and number of household members are encoded by a continuous numeric value. The average monthly household income (ainc) is valued between a minimum 17 (below 170,000 KRW) and a maximum 900 (above 9,000,000 KRW). The number of household members (cfam) is represented numerically with a maximum six members. Third, all the remaining SES features are categorical, which are encoded as in Table 1 for each category: urban/rural divisions (town_t); type of dwelling, i.e., apartment or non-apartment (apt_t); housing type (live_t); income quartiles for both individuals (incm) and households (ho_incm); income quintiles for individuals (incm5) and households (ho_incm5); educational levels (edu); and detailed educational classifications (educ).

### 4.2. Features of Social Infrastructure

Social infrastructure is important for sustaining muscle health in the senior community, as social facilities provide exercise programs and places for PA and opportunities for social interaction, which can enhance individual QoL.

In the infrastructure data denoted in the Participants and Data Selection section, we categorize social infrastructure into two groups that are related to sarcopenia. The first group includes social facilities, such as a cultural facility (cul_dist), social welfare facility (soc_dist), and senior leisure welfare facility (elder_dist). Cultural facilities include public libraries, museums, art galleries, cultural and art halls, local cultural centers, and cultural houses. Social welfare facilities include local centers conducting social welfare services for the aged, disabled persons, and children. Senior leisure welfare facilities include local centers that are more targeted to the welfare and leisure of the elderly rather than other ages and that provide specialized programs and consulting services for the health of the elderly. The second group includes urban park areas (park_area) and neighborhood sports facility areas (pa_area), which are public parks with a sport facility.

For infrastructure features, we differently preprocess two types of data, i.e., facility accessibility and per capita facility area. For the first group, we approximate the average distance (m) to the facility using the formula $\sqrt{\frac{\mathrm{residential}\;\mathrm{area}\;(m{}^{2})}{\mathrm{count}\;\mathrm{of}\;\mathrm{facility}}}$, considering the residential area as the region where the majority of the population lives. For this, we calculate the area of the residential sector in the urban area consisting of green, residential, commercial, and industrial sectors according to the urban planning status statistics published by the MOLIT [71], addressing that over 90% of South Korea’s total registered population resides in urban areas.

In the second group consisting of parks, we consider the regionally available infrastructure area per person and calculate the per capita facility area using the formula $\frac{\mathrm{facility}\;\mathrm{area}\;(m{}^{2})}{\mathrm{count}\;\mathrm{of}\;\mathrm{population}}$, in which the unit of the urban park area is 1000 m${}^{2}$, and the unit of the neighborhood sports facility area is also m${}^{2}$ in this study.

For this preprocessing, some of the population and facilities in the nonurban or nonresidential areas are missed. Therefore, it is more appropriate to understand facility accessibility features as encompassing general trends and information from densely populated urban areas rather than the entire country’s comprehensive situation. Nonetheless, the derived features are eligible, as the majority of the population is used for the calculation.

### 4.3. Features of Quality of Life

QoL is essentially related to satisfaction derived from material, social, and psychological experiences, greatly shaped by individual perspectives and evaluated through diverse factors. Among them, health status, including sarcopenia, can significantly impact QoL.

The EuroQol [33] questionnaire is designed as a QoL assessment tool, providing a comprehensive and standardized way of generating a numerical index that represents health condition. The EQ-5D-3L is a three-level version of the EQ-5D and is one of the most widely employed tools, which consists of two components, namely, a descriptive system and a visual analogue scale (VAS). The descriptive system includes survey questions that depict different health aspects across five dimensions: mobility (LQ_1EQL), self-care (LQ_2EQL), usual activities (LQ_3EQL), pain/discomfort (LQ_4EQL), and anxiety/depression (LQ_5EQL). Respondents can choose from three possible answers, i.e., “no problems”, “some problems”, and “extreme problems”, which are indicated by labels 1–3. Additionally, the visual analogue scale (LQ_VAS) records the respondents’ self-assessment of their own health on a vertical VAS, with the endpoints labeled as “best imaginable health” and “worst imaginable health”. This information can be used as a quantitative measure of individually assessed health outcomes, represented as a continuous value. More detailed information can be accessed through the EQ-5D guidelines [72].

In addition, the average daily sleep duration (BP8) and the perceived level of stress in daily life (BP1) are measured for the psychological domain as an important part of QoL. These are represented by continuous units of time and a segment with a scale of 1–4. Higher values on the stress scale indicate lower levels of perceived stress.

### 4.4. Statistical Analysis of Features

In this section, we conduct a statistical analysis of the values of the SES, social infrastructure, and QoL features in addition to the basic health information features, such as age, waist circumference (HE_wc), and body mass index (HE_BMI), according to the presence of sarcopenia. Additionally, we compare the statistics between males and females.

Table 2 and Table 3 present the statistic details of the selected features, excluding some categorical variables like (town_t, apt_t and live_t) because a statistical analysis of them could lead to ambiguous statistical interpretations due to a lack of meaningful ordinal relationships. It is worth noting that the observed differences in the average values are descriptive rather than causal, which are induced from various factors. Accordingly, a conclusion regarding specific individuals cannot be drawn solely based on these trends within the study population.

#### 4.4.1. Comparison by Gender

For features with *p*-values lower than 0.05, the statistical difference is observed according to gender. ASMI, as a key factor for sarcopenia diagnosis, is higher in males due to gender-related physiological characteristics. Moreover, HE_wc is higher in males, whereas HE_BMI is higher in females. In the binary features, gender-related differences are observed in EC1_1, allownc, house, genertn, and marri_2, except marri_1. On average, males are more likely to be engaged in economic activities, own homes, and live with a spouse. However, conversely, females receive more basic livelihood allowances, and they are more likely to be in one-person households without a spouse or family. In categorical features, differences between the two gender groups are observed in SES features, such as cfam, ho_incm, ho_incm5, edu, and educ. Males tend to have slightly larger family sizes and higher income quartiles and quintiles for the household. Educational factors also show a noticeable gender gap, with males having higher average values. All the LQ_1EQL to LQ_5EQL items in the QoL features show differences, where lower values indicate fewer disruptions in life. In all these items, the average values are lower for males, indicating that females experience more disruptions in their lives. LQ_VAS also displays a noticeable preference for males. While the average daily sleep duration as BP8 exhibits similar mean values, males have a slightly higher average. In the case of the stress index at BP1, in which higher values indicate lower stress, females show higher stress index values.

#### 4.4.2. Comparison by Sarcopenia Diagnosis

For each gender, we evaluate the features that demonstrate statistical significance on sarcopenia prevalence by employing a chi-square test for binary and categorical features and independent-samples *t*-tests for continuous features. A *p*-value below 0.05 indicates a notable difference in terms of sarcopenia prevalence.

Several notable findings are observed in the male population. First, individuals with sarcopenia tend to be older and exhibit lower ASMI values. Similarly, both HE_wc and HE_BMI demonstrate significant differences that individuals with sarcopenia have notably lower values. Among the SES features, EC1_1, ainc, ho_incm, ho_incm5, edu, and educ exhibit differences between groups with and without sarcopenia. This suggests that individuals with sarcopenia tend to have no economic activity and lower monthly household income. Additionally, this difference is also reflected in income quartiles and quintiles for the household. In terms of educational features, individuals without sarcopenia tend to have higher educational achievements. Furthermore, higher values of LQ_1EQL through LQ_5EQL and lower LQ_VAS scores in individuals with sarcopenia indicate that overall life satisfaction is lower among individuals with sarcopenia compared to the other group.

Similar trends are observed in the female population. It is evident that individuals with sarcopenia are generally older. ASMI values are also naturally lower, with the mean difference being smaller compared to males. HE_wc and HE_BMI also show significant differences, with much lower values compared to healthy individuals. Within the SES features, differences are observed in EC1_1 and genertn, but statistically significant differences are found in relatively fewer features compared to males. This implies that individuals with sarcopenia are relatively less economically active. Interestingly, individuals without sarcopenia appear to be more likely to live in households without a spouse or family members. In the QoL features, only LQ_1EQL exhibits group differences. In this item concerning self-care (whether there is difficulty in bathing or dressing alone), life satisfaction is lower among healthy individuals.

As a consequence, it has been observed that more diverse features show statistically significant differences based on the presence of sarcopenia, primarily in males rather than females. To better understand these factors, further investigations and additional epidemiological studies in different datasets may be required.

#### 4.4.3. Comparison by Urban and Rural Areas

To account for the annual variations in diverse residential area sizes and populations within each region, we divide the regions in South Korea into rural areas and urban areas. The urban areas include metropolitan cities and densely populated provinces such as Gyeonggi-do, while rural areas comprise the rest. When represented by each city code, numbers 1–8 correspond to urban areas, while numbers 9–16 represent rural areas. Table 3 compares the rates of sarcopenia and social infrastructure features between rural and urban areas.

When comparing the rates of sarcopenia between rural and urban areas, the urban areas exhibit a slightly higher rate (32.1%) compared to the rural areas (30.3%). The accessibility of cul_dist and elder_dist is significantly lower in urban areas than in rural areas. Only soc_dist shows a slightly better accessibility in urban areas. The results of park_area and pa_area indicate that urban areas have a significantly smaller per capita area for urban park and neighborhood sports facilities compared to rural areas. The tendency for urban areas to exhibit unfavorable feature values compared to rural areas might be attributed to the high population density and the consequent expansion of residential spaces.

These results highlight the overall differences in social infrastructure and environment between rural and urban areas, implying a potential correlation with the prevalence of sarcopenia. However, it is worth noting that the measured features do not account for transportation infrastructure, making accessibility and convenience distinct concepts.

## 5. Sarcopenia Prediction Model Using Machine Learning

Based on the aforementioned SES, social infrastructure, and QoL features that can be obtained by using a simple self-survey without expert assistance, we construct a predictive model for sarcopenia in the elderly population. Among the various learning algorithms, we select two gradient boosting models (LGB and CAT), one ensemble model (RF), and a neural network-based model (e.g., DNN).

LGB [73] is an ML algorithm based on gradient boosting trees and is known for its high performance and fast training speed. It operates efficiently even with large datasets and improves the accuracy by dividing data into arbitrary distributions for learning.

CAT [74] is also an algorithm based on a gradient boosting tree and is specialized in handling categorical variables, automatically dealing with categorical variables, and seeking optimal splits. It includes methods to prevent overfitting and improve training speed, making it user-friendly and convenient to use.

RF [75] is an ensemble technique that combines multiple decision trees, where each decision tree learns independently by using subsets of data, and the final prediction is determined by averaging or majority voting among them. It can be applied to various data types and sizes, reducing overfitting and providing stable predictions.

A DNN [76] is an artificial neural network that consists of multiple hidden layers. It is effective in extracting features from complex data and solving intricate problems. It can be applied in various fields, such as computer vision and natural language processing, and excels in tasks that require vast amounts of data and computational power to enhance performance.

The choice of LGB, CAT, RF, and DNN among various algorithms stems from their common strengths. These algorithms exhibit excellent predictive performance and provide support for a wide range of data types. They offer the flexibility to adjust model complexity according to the characteristics of the data, preventing overfitting. Additionally, they deliver efficient processing speeds and various methods for enabling model interpretability. They are versatile tools widely used in the data science ecosystem, applicable across diverse domains and applications.

### Explainable AI: SHAP and Causal Inference

To investigate the prediction models using explainable artificial intelligence (XAI), we introduce a SHAP value to derive the feature importance against the model outcomes. Furthermore, causal inference is performed to ascertain the causality between sarcopenia as the outcome and a specific feature as the cause.

There are methods for calculating feature importance for each of the mentioned algorithms. However, these methods can vary in how they calculate importance due to the specific workings of each algorithm. Additionally, in the case of ensemble models where multiple models are combined, the results can lack consistency. Notably, in tree-based models, randomness can be introduced based on the model’s structure and input data. On the other hand, SHAP values explain the influence of features for individual predictions. SHAP calculates the importance of all features mathematically, providing accurate and consistent results independent of the model type or structure. These characteristics enhance the interpretability of predictions and contribute to increased trust in the results.

An analysis of the SHAP value can be used to interpret and explain the prediction results of ML models, quantifying how much each feature contributes to the model’s predictions. It provides a quantitative value of the relative importance of each feature and their impact on the model’s predictions. The core of the SHAP analysis lies in the concept of Shapley values [38,39] that originate from cooperative game theory and involve distributing the contributions of various features fairly, considering their interactions. In this study, the widely used and comprehensive SHAP library [37] is employed, providing a range of functionalities for interpretation and analysis. Through this, we aim to identify which features play a crucial role in the prediction of sarcopenia.

Causal inference is a statistical analysis and inference domain employed to understand and explain the relationship between causes and their corresponding effects. Recently, methods that explain causation using solely observed data have garnered attention as alternatives to experimental approaches, such as costly randomized controlled trials (RCTs). In causal inference, methods such as a directed acyclic graph (DAG) and potential outcomes calculate the average treatment effect (ATE) by computing the average difference in outcomes between the treatment and control groups. For instance, in binary treatment, the treatment group consists of individuals with a value of 1, indicating that they belong to a specific treatment, while the control group is composed of individuals with a value of 0. For continuous treatment, groups are made according to the numerical value of the treatment. For instance, we can designate the third quartile value of the treatment variable as the treatment group, while we can set the remaining first quartile value as the control group. In the case of binary treatment, the ATE originally represents E[Y(1)−Y(0)]. Here, *Y* represents the outcome, meaning that Y(T) denotes the outcome when the subject is intervened with to take a specific treatment *T*. However, because we cannot directly measure the counterfactual from the observed data, we average out each potential outcome, respectively, which leads to E[Y(1)]−E[Y(0)].

The ATE, calculated as the difference in outcomes between the treatment and control groups, quantifies the effect of the treatment. However, because the ATE calculates the average effect of treatment across all individuals, the impact of treatment may not be consistently observed across individuals. In such cases, using the CATE as a metric to evaluate the TEs based on specific conditions or characteristics can yield more meaningful results. Accordingly, the CATE overcomes the inconsistencies of the ATE for individuals by accounting for the diversity of TEs, and it provides more accurate estimates of the effects under specific conditions. For this, it is crucial to identify and consider the confounding variables, which are external factors that can distort or mislead causal relationships. This enhances our understanding of the TEs and their variations across different contexts.

Because the RCTs and DAGs suffer often from high costs when dealing with a large number of variables, we opt for the potential outcome framework as a causal inference method that we consider more suitable for our environment. Among these, double machine learning (DML) stands out for its unique capabilities. Unlike certain meta-learners that are restricted to discrete treatments, DML can be effectively applied to scenarios involving continuous treatments. Additionally, it excels when dealing with a multitude of confounding variables. Hence, we have chosen the DML approach within EconML [77], allowing us to estimate the CATE θ(X) based on the following structural equation (Equation (1)). Here, *T* represents the treatment, *X* represents the variable for which we want to determine the effect of the treatment on the outcome, *W* represents the confounding variables, and *Y* represents the outcome. DML is particularly attractive as it refrains from imposing additional structural assumptions on *g* and *f* by nonparametric ML techniques.

(1) $$\begin{array}{cc} Y & =\theta (X)\cdot T+g(X,W)+\epsilon \;T=f(X,W)+\eta \\ E & [\epsilon |X,W]=0\;E[\eta |X,W]=0\;E[\eta \cdot \epsilon |X,W]=0 \end{array}$$

DML is based on the regression concept used when there are two sets of variables, $X_{1}$ and $X_{2}$. In this example of estimating linear regression model parameters, $\beta_{1}$ and $\beta_{2}$, for the equation $Y=\beta_{1}X_{1}+\beta_{2}X_{2}$, we follow these steps to obtain the $\beta_{1}$ set:

1. First, perform a regression of *Y* on the second set of features, resulting in $Y_{1}^{r}=\gamma_{1}X_{2}$.
2. Next, conduct a regression of the first set of features on the second set of features, which yields $X_{1}^{r}=\gamma_{2}X_{2}$.
3. Then, calculate the residuals $Y^{r}=Y-Y_{1}^{r}$ and $X^{r}=X_{1}-X_{1}^{r}$.
4. Finally, regress the residuals of the outcome ($Y^{r}$) on the residuals of the features ($X^{r}$). This regression results in $Y^{r}=\beta_{1}X^{r}+\alpha$.

By these steps, we obtain the same $\beta_{1}$ as we would obtain by regressing all the features together. To calculate $\beta_{2}$, we can follow the same steps in reverse while focusing on the relationship between *Y* and the second set of features, $X_{2}$. In the above regressions, the first set of features can represent our treatment, while the second set of features can serve as confounding factors so that we can separately estimate the impact on the outcome.

DML employs the straightforward concept as explained above but uses ML models instead of linear regression, allowing it to handle non-linearity and interactions when estimating the treatment and outcome residuals. For this, we first need to derive E[Y∣X,W] to express structural equations differently, which is the conditional expected value of *Y* given the conditions of the provided *X* and *W* as follows:

(2) $$\begin{array}{cc} E[Y|X,W] & =E[\theta (X)\cdot T+g(X,W)+\epsilon |X,W]\\ \; & =\theta (X)\cdot E[T|X,W]+g(X,W) \end{array}$$

We reformulate Equation (1) by subtracting Equation (2) as follows:

(3) $$\begin{array}{cc} Y-E[Y|X,W] & =(\theta (X)\cdot T+g(X,W)+\epsilon )\\ \; & -(\theta (X)\cdot E[T|X,W]+g(X,W))\\ Y-E[Y|X,W] & =\theta (X)\cdot (T-E[T|X,W])+\epsilon \end{array}$$

Because we can realize the conditional expectation functions as ML regression tasks from the given data, i.e., q(X,W)=E[Y∣X,W] and f(X,W)=E[T∣X,W], then we can express the outcome through the following equation:

(4) $$\begin{array}{cc} \tilde{Y}=\; & Y-q(X,W)\\ \tilde{T}=\; & T-f(X,W)=\eta \\ \tilde{Y}=\; & \theta \left(X\right)\cdot \tilde{T}+\epsilon \end{array}$$

Therefore, given that E[η·ϵ∣X]=0, the estimation of θ(X) becomes a final regression problem as in Equation (5) involving $\tilde{Y}$ with *X* and $\tilde{T}$, although being limited to models that are linear in $\tilde{T}$.

(5) $$\begin{array}{c} \hat{\theta }=\arg\min_{\theta \in \Theta }E_{n}\left[{\left(\tilde{Y}-\theta \left(X\right)\cdot \tilde{T}\right)}^{2}\right] \end{array}$$

Through this process, we can determine the CATE on the variable X for causal relationships, which helps in revealing more precise causal relationships. Although a particular correlation indicates a high statistical association between two variables, it does not inherently imply a causal relationship. The causation signifies a relationship that one variable influences the outcome of another. An experimental design or intricate statistical analysis is required to ascertain causation. Relying solely on correlation makes it difficult to accurately comprehend the cause-and-effect relationship between variables. Through causal inference, we aim to discern the influence of various features on sarcopenia.

## 6. Experiments

We use the aforementioned ML algorithms to train models for predicting sarcopenia. Each model is trained using male-only samples, female-only samples, and both genders to investigate the potential difference in performance according to gender. We conduct a stratified fourfold cross-validation to assess the models’ general performance using the entire provided dataset for training and validation. This procedure involves utilizing 75% of the data as training samples and 25% as validation samples for each fold. The final result is presented as the average value obtained from the cross-validation iterations. Stratified K-fold cross-validation is one of the cross-validation techniques used primarily in classification problems. Instead of randomly dividing the data, this method ensures that the ratio of classes (labels) is maintained consistently across each fold. This prevents data imbalances or biases by keeping the class proportions uniform in each fold. This approach allows for a reliable performance evaluation of how the model generalizes to the entire dataset. Moreover, by calculating the average across K folds, it reduces variance and enhances the model stability.

RobustScaler is used to perform data normalization in sklearn library [78] for all features and is particularly suitable for medical data characterized by numerous outliers. From experiments with other normalization methods such as StandardScaler and MinMaxScaler, it was observed that RobustScaler ultimately achieved the highest accuracy. It adjusts data by considering the median and the interquartile range (IQR) spanning from the 25th to the 75th percentile of the data distribution. As a result, it can facilitate the extraction of common patterns from datasets containing outliers.

Moreover, we conduct hyperparameter tuning for each model; however, as we already observed in our previous study [47], the performance enhancement is marginal compared to the outcome from the default configuration on each model.

### 6.1. Accuracy of Prediction Models

First, we aim to compare the accuracy by using various feature combinations. In addition to Basic features, we measure the accuracy with more learning features of the SES, social infrastructure (Infra), and QoL. Table 4 shows the accuracy results of the additive features from the SES to the QoL to the Infra as an order. Other combinations are omitted due to space, but the results can be similarly expected as follows.

For only the Basic features, all the models except for DNNs achieve an accuracy of over 75%. Especially, CAT outperforms with the highest accuracy, recording 76.9% for both genders, 78.0% for males, and 78.8% for females. With the SES features in addition to the Basic features, all the models achieve improvement in accuracy. In both genders, RF shows the highest at 78.3%, while CAT retains the lead of 78.3% and 79.6% for males and females, respectively. Employing the Basic, SES, and QoL features, CAT continues to mark the highest accuracy, albeit not exceeding 80%. With the incorporation of the Infra features, the accuracy becomes less or comparable than the previous case as CAT achieves only 74.9% for both genders and the accuracy for each gender is 78.3 and 74.6%. From the results, we can conjecture that the Infra features are too complicated to describe the features based on the distance or area of the facilities for ML with limited samples.

### 6.2. Confusion Matrix of Prediction Models

**Figure 2:**
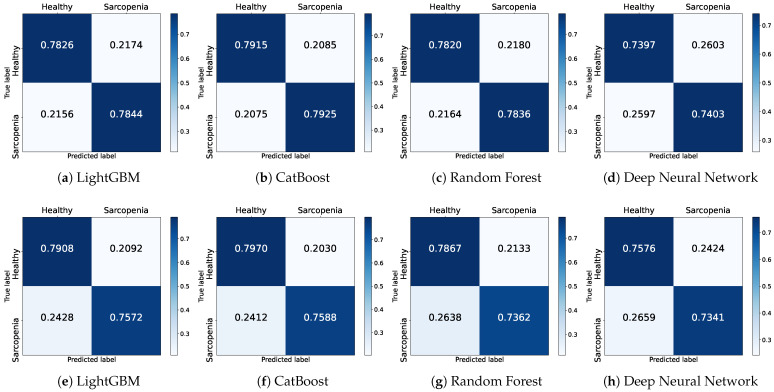
Confusion matrices of prediction models using the male (**a**–**d**) and female (**e**,**f**) datasets with Basic + SES + QoL features.

The confusion matrix is a table extensively used in ML to evaluate the performance of classification models and provides a comprehensive breakdown of the predicted outcomes versus the actual class labels. The confusion matrix consists of four key elements. Accurate predictions for the positive and negative classes are labeled as true positive (TP) and true negative (TN), respectively. An incorrect prediction, where a negative is classified as positive, is labeled as false positive (FP). Conversely, when a positive is wrongly classified as a negative, the prediction is labeled as false negative (FN). Thereby, the confusion matrix offers valuable insights into the strengths and weaknesses of the classification model. In our study, sarcopenia is considered as the positive class, while healthy individuals are considered as the negative class. Figure 2 shows the results for the Basic + SES + QoL features, which achieved the highest accuracy. In the figure, the row values are normalized to 1 to calculate the models’ prediction ratio.

Figure 2a–d illustrate the confusion matrices of the four models trained on the male dataset. LGB correctly predicts healthy individuals by approximately 78.3% while misclassifying by 21.7%. Furthermore, it correctly classifies actual sarcopenia patients by 78.4% and misclassifies by 21.6%. CAT achieves the highest classification rates for healthy individuals and individuals with sarcopenia at 79.2% and 79.3%, respectively. Similar to LGB, in the RF model, the proportion for correctly classified healthy individuals and individuals with sarcopenia is 78.2% and 78.4%, respectively, indicating a comparable performance. The DNN model also demonstrates results with an accurate classification ratio of 74.0% and 74.0% for each class. In the confusion matrices, the models, except for the DNN, exhibit similar classification performance.

Figure 2e–h illustrate the confusion matrices of the four models trained on the female dataset. Overall, the prediction ratios for healthy individuals are similar to the male dataset. However, the correct classification ratio for sarcopenic individuals has decreased. The correct prediction ratios for individuals with sarcopenia decrease by approximately 3% for LGB and CAT and by a significant 5% for RF. The DNN shows a slight 1% decrease compared to the previous results.

### 6.3. Precision and Recall of Prediction Models

Precision and recall values can be investigated based on the confusion matrix. Precision represents the ratio of TP cases among those predicted as positive by the model, $\frac{\mathrm{TP}}{\mathrm{TP}\;+\;\mathrm{FP}}$, highlighting the importance of accurate positive predictions against false alarms. Conversely, recall indicates the ratio of TP cases among all actual positives, $\frac{\mathrm{TP}}{\mathrm{TP}\;+\;\mathrm{FN}}$, focusing on minimizing the instances of missed positive cases as sensitivity. In summary, precision assesses how accurately the model predicts positives, while recall assesses how effectively the model detects actual positives. The results for precision can be seen in Table 5, and the results for recall are available in Table 6.

In the Basic features, excluding the DNN models, a precision of over 0.75 in all the models is achieved. Notably, CAT demonstrates the highest precision with 0.767 for both genders, 0.784 for males, and 0.758 for females. With the integration of the SES feature into the Basic features, there is an enhancement in precision for all the models, except CAT and the DNN for males. For the group with both genders, RF tops the list with a precision of 0.776, whereas for males and females, CAT continues to lead with 0.782 and 0.767, respectively. Employing the Basic, SES, and QoL features, CAT does not exceed 0.80, but it records the highest values among the experimented feature combinations. As more features are introduced, some models exhibit slight fluctuations in performance, but most observe an improvement compared to their predecessors. After integrating the Infra features, the highest precision decreases to 0.752 for both genders, 0.785 for males, and 0.723 for females, which is lower than the previous case.

Utilizing only the Basic features, all the models, excluding the DNN, achieve a recall of over 0.75. Among them, CAT showcases the highest recall with 0.769 for both genders, 0.782 for males, and 0.788 for females. The integration of the SES feature into the Basic features leads to an enhancement in recall for the majority of models. For the group with both genders, RF takes the lead with a recall of 0.783, while for males and females, CAT persistently dominates with recalls of 0.781 and 0.796, respectively. When employing the Basic, SES, and QoL features, CAT does not exceed 0.80, but it records the highest values among the experimented feature combinations. As more features are incorporated, slight performance variations are observed in some of the models, but the majority show an increase compared to before. After integrating the Infra features, the highest recall decreases to 0.748 for both genders, 0.783 for males, and 0.740 for females, which is lower than the previous case.

The precision and recall exhibit similar trends. Particularly, CAT consistently demonstrates the highest precision and recall values across various feature combinations, irrespective of gender. LGB and RF also showcase values proximate to CAT, while the DNN lags behind with relatively lower figures. Introducing other features alongside the Basic features results in performance enhancements, with the combination of the Basic + SES + QoL features achieving the highest values. This suggests that the SES and QoL features play a pivotal role in augmenting the predictive prowess of the model. Unfortunately, with the incorporation of the Infra features, most of the models fail to sustain their previous performance, which implies that the addition of Infra features might not substantially benefit most models in terms of precision and recall.

### 6.4. Performance of Prediction Models

**Figure 3:**
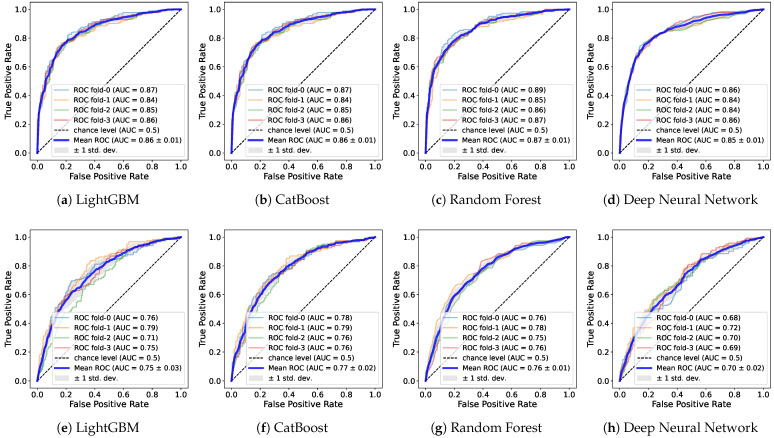
ROC curves of prediction models using the male (**a**–**d**) and female (**e**,**f**) datasets with Basic + SES + QoL features.

The ROC and AUC are pivotal metrics used to evaluate the overall performance of binary classification models. The ROC curve offers an inclusive performance assessment even in scenarios with a minority class. The AUC refers to the area under the ROC curve that plots a model’s true positive rate (y-axis) and false positive rate (x-axis) at various thresholds, as shown in Figure 3, which displays the ROC curve of the model results for the Basic + SES + QoL features. Here, a higher AUC value, closer to 1, indicates superior performance of the model in class imbalances. Consequently, through the AUC, an evaluation that takes into account class imbalances is attainable. The results for the AUC are presented in Table 7.

Utilizing the Basic features, only LGB and CAT surpass a value of 0.81 for both genders. For males, all the models exceed 0.8, with CAT standing out with a peak value of 0.858. In contrast, for females, the highest value is 0.767 by CAT, and the DNN notably records a significantly low value of 0.678. When the SES is integrated into the Basic features, RF also exceeds 0.81 for both genders along with LGB and CAT. For males, all the models surpass 0.81, and for females, all the models achieve over 0.7. The SES improves the performance across all the models. With the Basic + SES + QoL features, the majority show an enhancement in performance, 0.82 for both genders and 0.85 for males. For females, the results remain relatively unchanged from previous combinations. Similar to the aforementioned result, there is performance degradation for all the features including the Infra features across all the models.

Consequently, the CAT model mostly outperforms regardless of the features and genders. Specifically, as the features are incrementally added, it accomplishes an AUC from 0.824 to 0.831 for both genders and from 0.858 to 0.868 for the male group. In the female group, it seems the influence of the QoL features is less pronounced, with the highest score of 0.775 recorded in the Basic + SES features. In terms of genders, discernible performance disparities are observed when the genders are delineated. Regardless of the feature combinations, performance improvements are consistently pronounced for males rather than for both genders, whereas there is a decline for females. Such outcomes suggest that the features selected for training in this study are potentially more favorable for the male dataset. Hence, training gender datasets separately could further optimize performance.

#### Performance of Prediction Models with Oversampling

With imbalanced datasets, the learning process tends to neglect the minority class, which can lead to degradation in predictive performance for that minority class. For this, several oversampling techniques have been proposed. The Synthetic Minority Oversampling Technique (SMOTE) [79] is one of the most widely adopted methods, aiming to artificially augment the number of instances in the minority class, which potentially facilitates better model training on the minority class for performance improvement. The SMOTE synthesizes new instances by leveraging the feature space between existing minority samples; specifically, a random minority instance is selected, followed by a random pick from its k-nearest neighbors. The difference between these two instances is computed and multiplied by a random value between 0 and 1 for the new instance.

In this section, we amplify the count of the minority class, i.e., sarcopenia samples, by using the SMOTE and compare with our previous results on the imbalanced samples. After partitioning the data into training and validation sets for a stratified K fold, oversampling is applied exclusively to the training set’s minority class. Table 8 shows the AUC results on a balanced dataset by the SMOTE.

In the Basic features, most models experience a slight decline in the AUC values. With the addition of the SES features, a consistent decrease is observed in both genders and females, while in the male group, all the models except CAT exhibit a slight increase. Similarly, performance with the Basic + SES + QoL features decreases mostly, except in the case of LGB for females, registering an increase up to 0.753. However, a relatively significant performance enhancement is observed for all features, including the Infra features. For both genders, LGB, CAT, and RF surge to respective values of 0.773, 0.792, and 0.778, marking a notable ascent. Most of the models exhibit growth, for example, RF jumps from 0.639 to 0.679 especially in the females rather than the males. The results from the oversampling fail to display any significant performance enhancement, although a modest yet noteworthy ascent is discerned for the Infra features. Therefore, we can conjecture that the one model trained by the original dataset is not biased and robust for classification compared to the other trained by the oversampled dataset.

As an overall summary, the proposed model achieves maximum AUC values of 0.831, 0.868, and 0.773 for both genders, males, and females, respectively, with the Basic + SES + QoL features. From the model perspective, LGB, RF, and CAT are comparable, but the DNN performs relatively lower. Moreover, as the utilized features especially bolster performance pertaining to males, segregating by gender and tailoring model training accordingly might prove advantageous. Such a prediction outcome is comparable with the aforementioned sarcopenia studies on the KNHANES dataset.

## 7. Discussion

### 7.1. SHAP Analysis

**Figure 4:**
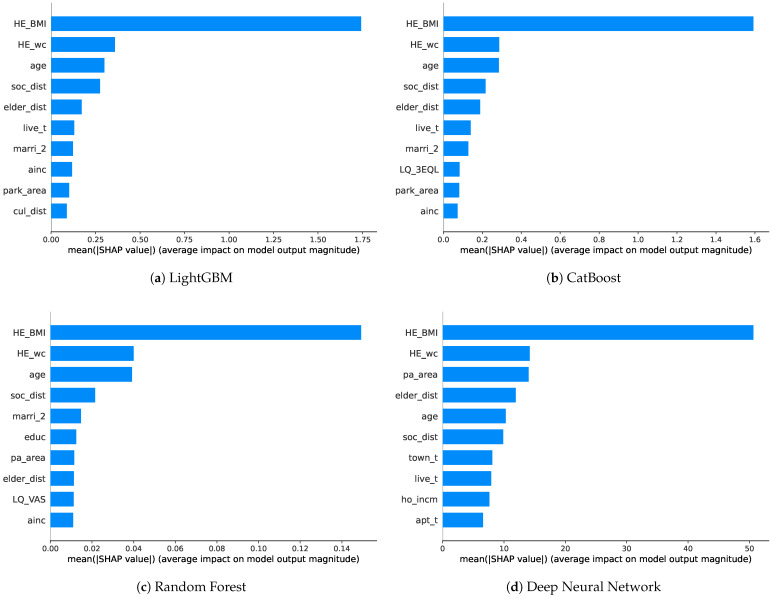
SHAP plots for top ten feature importances of prediction models.

Through the SHAP values, the general influences of features are investigated from both genders and graphically visualized as to how each feature impacts the models’ outcomes. Figure 4 shows the SHAP values for each model. We then measure the frequency of the top 10 features with the highest SHAP values in each model and score them to identify common factors. The features commonly used across the models can be regarded as relatively more effective in predicting sarcopenia, among other numerous features employed in this study.

The boosting models (LGB and CAT) exhibit similar orders of SHAP values. HE_BMI stands out as the most crucial feature, followed by HE_wc, age, soc_dist, and elder_dist in the top five. The ordering remains consistent up to live_t and marri_2, with differences in the subsequent rankings. Indeed, ainc and park_area are included, while cul_dist appears in LGB and LQ_3EQL appears in the CAT models. Similarly, for RF, HE_BMI overwhelmingly ranks first in importance, followed by HE_wc, age, and soc_dist in the same order as the boosting models. However, previously unseen features, such as educ, pa_area, and LQ_VAS, emerge afterward. The DNN presents different rankings from the previous models. HE_BMI and HE_wc are similar to the other models; however, pa_area and elder_dist are positioned slightly above age. The subsequent features include the new town_t, ho_incm, and apt_t. In the case of the DNN, the SHAP values are more evenly distributed compared to the other models.

It can be challenging to directly compare the mean SHAP values due to their own characteristics across different models. Instead, a plausible approach to score the top 10 most significant features used in well-performing models can be considered. This approach allows for the concise identification of common important features, with feature scores presented in Table 9, ranging from 0 to 4, which are calculated by summing the values assigned to the used features (marked as 1) and unused features (marked as 0) for each model.

Across all the models, age, BMI, waist circumference, accessibility to social welfare facilities, and accessibility to senior leisure welfare facilities are the features with SHAP values that received a score of 4. These features are considered to have the most significant impact on predicting sarcopenia outcomes. Following these, monthly average household total income, housing type, and marriage status receive a score of 3. With a limited range of models tested, we focus on the features that received scores above a score of 3. All the mentioned features that received high scores, including the Basic, SES, and social infrastructure features, can be considered as the most crucial in predicting sarcopenia, on average.

### 7.2. Causality Analysis

The CATE results provide insights into the causal relationships between various features and the occurrence of sarcopenia. However, when attempting to investigate the causal relationship between the treatment (the specific feature of interest) and sarcopenia, it is unclear which features need to be configured as confounding variables and measured for their influence. Hence, we use all the features besides the treatment, influencing both the outcome and the treatment for the CATE calculation (also including the gender feature). In addition, age has been selected as a conditional variable for the above calculation, considering that sarcopenia is strongly associated with age, and features other than age may be intractable for intuitive interpretation. Consequently, we analyze how specific treatments interact with sarcopenia with respect to age, comparing the TE by applying the maximum treatment value to the treatment group and the minimum value to the control group. As the outcome is defined as 1 for sarcopenia and 0 for normal, a negative TE implies a decrease in risk for sarcopenia, while a positive TE indicates an increase in risk for sarcopenia.

For the causal analysis, only four selected features are introduced separately from each category. These features include the SES, social infrastructure, QoL, and basic information, which relatively shows a remarkable trend for the causal relationship compared to the other features within the category.

**Figure 5:**
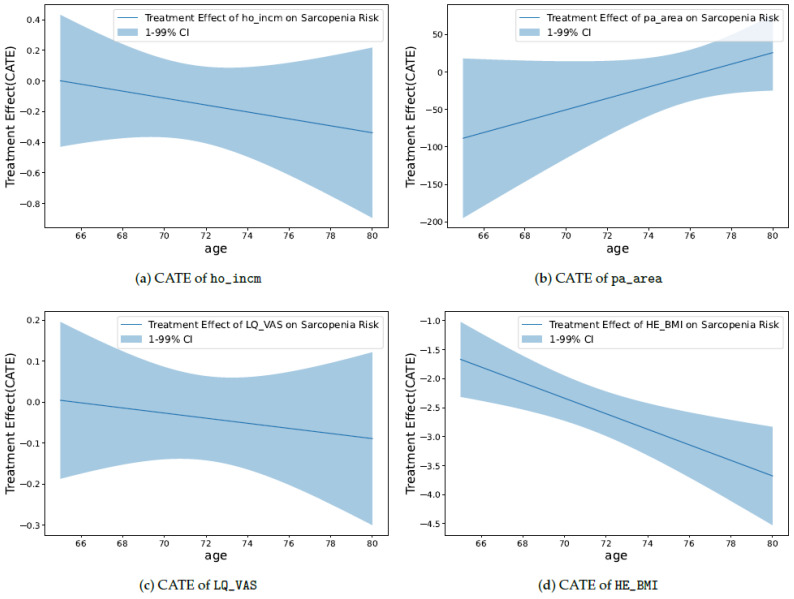
CATE plots of specific treatment on sarcopenia risk.

As shown in Figure 5a, the initial TE of ho_incm starts from 0 and gradually decreases. When individuals are relatively young, the impact of a TE seems minor. However, as age increases, the high-income quartile for households is associated with a reduced risk of sarcopenia. Furthermore, as age increases, the TE becomes more negative, indicating a strengthened effect. In summary, a higher-income quartile for households corresponds to a reduced risk of sarcopenia. This effect intensifies with age.

As shown in Figure 5b, pa_area starts with a relatively large negative value compared to other plots. This indicates a significant association between a large per capita area of neighborhood sports facilities and a reduced risk of sarcopenia. However, as age increases, this effect gradually diminishes, with the TE approaching closer to 0. In other words, a larger per capita area for neighborhood sports facilities is linked to a reduced risk of sarcopenia; however, this effect decreases with increasing age.

As shown in Figure 5c, LQ_VAS starts with a TE value of approximately 0 and exhibits a slightly negative slope. This indicates that in younger groups, life satisfaction might not play a significant role. However, as age increases, high life satisfaction becomes associated with a reduced risk of sarcopenia. Moreover, the TE gradually decreases with age, suggesting that this effect becomes slightly more pronounced in older individuals. In essence, higher life satisfaction is linked to a reduced risk of sarcopenia. This effect is subtly reinforced with increasing age.

As shown in Figure 5d, the TE of HE_BMI is consistently negative from the start. This indicates that a high BMI contributes to the reduction in the risk of sarcopenia. Moreover, as the age increases, the TE becomes more negative, suggesting that the influence of BMI on sarcopenia intensifies with age. In other words, a higher BMI is associated with a reduced risk of sarcopenia. This effect becomes more pronounced with increasing age.

Through the aforementioned analysis, the causal relationships between those features and the risk of sarcopenia can be confirmed. In conclusion, factors, such as income quartile for households, per capita area of neighborhood sports facilities, life satisfaction, and BMI, play significant roles in reducing the risk of sarcopenia. Understanding these causal relationships empowers us to develop more precise strategies for prevention and management.

## 8. Limitations and Future Work

The results of the machine learning-based prediction of sarcopenia in this study could serve as a starting point for research in the direction of identifying high-risk groups for sarcopenia by using easily obtainable sociological information, without the need for specific screening tests. Through the development of models with sufficient data and validation, early diagnosis and treatment can become more accessible.

However, there are some limitations to be considered. First, the KNHANES data we used were obtained between 2008 and 2011, which are somewhat dated. This is because the necessary muscle-related data for diagnosing sarcopenia are not available in the subsequent years. Therefore, we are limited to using data from that specific period. However, it is expected that the results obtained would not significantly differ from those of other years. Nevertheless, there is an increasing need for collecting high-quality, up-to-date data.

Second, for facility accessibility, we use the values from the residential area within the sector classified as urban to compute this feature. However, because some facilities are distributed in nonurban or nonresidential areas, this does not take into account the radius to all facilities from every residential area in South Korea. However, it is worth noting that this reflects the overall trend in South Korea, where the majority of the population is concentrated in densely populated urban areas. Additionally, due to concerns over personal privacy, the dataset only provides information on metropolitan cities and provinces, not detailed city names (townships or neighborhoods). This encompasses a broader range than the actual vicinity in which individual participants reside and operate. Furthermore, accessibility may not be determined solely by distance to a facility. For example, although such a distance in the rural area is relatively shorter because of the small residential area compared to the urban area, the public transportation system is less competitive. As a result, due to the complexity of social infrastructure and difficulty in gathering information, a more specific and accurately measured dataset is required.

Thirdly, in the calculation of the CATE for the causal analysis, assumptions are made for all variables except the specific treatment of interest to estimate the TE. It is assumed that all variables, including gender as used in this study, influence both the treatment and the outcome. We make this assumption due to the lack of information from the observed data to determine the actual relationships. We assume interdependencies among various SES, social infrastructure, and QoL data and obtain the results. However, it should be acknowledged that these results may vary depending on different variable combinations or datasets.

In the future, we aim to establish a healthcare system that can proactively identify individuals at risk of sarcopenia by using these features that can be obtained from a simple survey, considering that sarcopenia is not widely recognized in Korean society, making self-awareness challenging for patients. For the infrastructure features, we plan to verify their influence in the machine learning domain integrated with future sarcopenia predictions through more detailed data collection. Additionally, we plan to develop a reliable prediction model by incorporating multimodal learning that takes into account a wide range of complex factors, as well as leveraging heterogeneous and oversampled datasets.

Furthermore, the insights gained from the analysis of the SHAP and causality will contribute to building the sarcopenia model and selecting potential features for its prediction. We will also explore the causal features of sarcopenia by using advanced models that align with the evolving field of causal inference. In particular, we use age as a conditional variable in our causal analysis. However, it would be meaningful to analyze the causality between sarcopenia and other conditional variables. Such an exploration of features is planned to be applied in future feature selection to enhance the efficiency of sarcopenia prediction. For verification of the proposed approach, we will evaluate our model with the available global datasets.

## 9. Conclusions

It is inevitable to explore sarcopenia not solely by the specific medical or physical conditions of individuals but also from a sociological perspective. In this study, we accordingly consider the SES, social infrastructure, and QoL factors that are measured more accurately due to their well-defined criteria and metrics to develop a sarcopenia prediction model. For comparison, we conduct experiments with various feature combinations.

We adopt the KNHANES data to compare the prediction performance and reliability with previous studies. As a result, we successfully identify sarcopenia with approximately 80% accuracy by using various ML techniques, such as RF, LGB, CAT, and a DNN. Moreover, despite class imbalance, AUC values of 0.831, 0.868, and 0.773 are achieved for both genders, males, and females, respectively. Compared with previous studies, these results show that various social factors can improve the performance across models.

According to the SHAP analysis, accessibility to social welfare facilities and accessibility to senior leisure welfare facilities are most commonly effective features in addition to the basic health information, such as age, BMI, and waist circumference. Additionally, the causal inference analysis reveals that high household income, life satisfaction, BMI, and the per capita neighborhood sports facility area play a positive role in reducing the risk of sarcopenia. It is worth noting that the per capita neighborhood sports facility area shows only a decrease in its influence as individuals age in contrast to others.

These findings can serve as a solid technical foundation for the proactive identification of individuals and groups at risk of sarcopenia. The early identification of at-risk individuals can play a pivotal role in the implementation of suitable treatments and interventions for the prevention of sarcopenia. In turn, this can facilitate future research and governmental efforts aimed at effectively managing sarcopenia. We will make an effort to enhance the model performance and reliability for the global elderly by using recent ML techniques, such as unsupervised and multimodal learning with heterogeneous and oversampled datasets.

## Figures and Tables

**Table 1:** Categorical features in socioeconomic status.

Feature	Code	Description
town_t	1	Urban Area
	2	Rural Area
apt_t	1	Non-Apartment Housing
	2	Apartments
live_t	1	Detached Houses
	2	Apartments
	3	Townhouses
	4	Multi-Unit Houses
	5	Other Types
incm, ho_incm	1	Lowest Quartile
	2	Lower-Middle Quartile
	3	Upper-Middle Quartile
	4	Highest Quartile
incm5, ho_incm5	1	Lowest Quintile
	2	Lower-Middle Quintile
	3	Middle Quintile
	4	Upper-Middle Quintile
	5	Highest Quintile
edu	1	Elementary School or Lower
	2	Middle School Graduate
	3	High School Graduate
	4	College Graduate or Higher
educ	1	Preschool
	2	Village School or Private Tutor
	3	No Formal Education
	4	Elementary School
	5	Middle School
	6	High School
	7	2-Year/3-Year College
	8	4-Year College
	9	Graduate School

**Table 2:** Statistics for Basic, SES, and QoL features.

Characteristics	Male (*n* = 1679)				Female (*n* = 2232)			
	**Total**	**Nonsarcopenia (** ***n*** **= 942)**	**Sarcopenia (** ***n*** **= 737)**	**p Value**	**Total**	**Nonsarcopenia (** ***n*** **= 1746)**	**Sarcopenia (** ***n*** **= 486)**	**p Value**
ASMI	7.14 ± 0.82	7.71 ± 0.55	6.41 ± 0.44	<0.001	5.92 ± 0.67	6.16 ± 0.54	5.06 ± 0.27	<0.001
Basic Features								
age	71.74 ± 4.57	70.75 ± 4.31	73.01 ± 4.58	<0.001	72.01 ± 4.69	71.70 ± 4.61	73.13 ± 4.81	<0.001
HE_BMI	23.08 ± 2.92	24.57 ± 2.42	21.18 ± 2.33	<0.001	24.12 ± 3.43	24.82 ± 3.31	21.60 ± 2.6	<0.001
HE_wc	84.53 ± 9.05	88.02 ± 7.72	80.06 ± 8.65	<0.001	83.16 ± 9.72	84.75 ± 9.41	77.44 ± 8.6	<0.001
Socioeconomic Status Features								
EC1_1	0.45 ± 0.50	0.49 ± 0.5	0.40 ± 0.49	<0.001	0.30 ± 0.46	0.31 ± 0.46	0.26 ± 0.44	0.037
allownc	0.07 ± 0.26	0.06 ± 0.25	0.08 ± 0.28	0.158	0.14 ± 0.34	0.14 ± 0.34	0.14 ± 0.35	0.812
house	0.84 ± 0.37	0.85 ± 0.35	0.82 ± 0.39	0.051	0.71 ± 0.45	0.72 ± 0.45	0.70 ± 0.46	0.547
genertn	0.07 ± 0.26	0.07 ± 0.25	0.07 ± 0.25	0.784	0.26 ± 0.44	0.27 ± 0.44	0.22 ± 0.42	0.035
marri_1	1.00 ± 0.04	1.00 ± 0.05	1.00 ± 0.04	0.712	1.00 ± 0.05	1.00 ± 0.04	1.00 ± 0.06	0.323
marri_2	0.91 ± 0.28	0.92 ± 0.27	0.91 ± 0.29	0.391	0.48 ± 0.5	0.48 ± 0.5	0.45 ± 0.5	0.245
ainc	164.34 ± 168.75	171.78 ± 164.74	154.84 ± 173.38	0.041	153.26 ± 184.37	149.49 ± 180.48	166.79 ± 197.31	0.082
cfam	2.59 ± 1.19	2.56 ± 1.17	2.61 ± 1.22	0.887	2.45 ± 1.39	2.40 ± 1.37	2.61 ± 1.44	0.099
incm	2.51 ± 1.11	2.57 ± 1.11	2.44 ± 1.11	0.121	2.49 ± 1.11	2.47 ± 1.11	2.53 ± 1.10	0.336
ho_incm	1.85 ± 1.00	1.94 ± 1.02	1.73 ± 0.96	<0.001	1.74 ± 1.00	1.73 ± 1.00	1.78 ± 1.02	0.746
incm5	3.01 ± 1.40	3.08 ± 1.41	2.93 ± 1.39	0.106	2.99 ± 1.41	2.97 ± 1.41	3.07 ± 1.41	0.318
ho_incm5	1.96 ± 1.26	2.06 ± 1.28	1.84 ± 1.22	0.002	1.84 ± 1.25	1.83 ± 1.24	1.89 ± 1.29	0.849
edu	1.92 ± 1.09	1.99 ± 1.10	1.83 ± 1.06	0.008	1.21 ± 0.60	1.21 ± 0.60	1.21 ± 0.60	0.948
educ	5.07 ± 1.51	5.17 ± 1.51	4.95 ± 1.50	0.019	3.85 ± 1.01	3.86 ± 1.01	3.82 ± 1.03	0.593
Quality of Life Features								
LQ_1EQL	1.33 ± 0.50	1.28 ± 0.47	1.41 ± 0.53	<0.001	1.58 ± 0.55	1.59 ± 0.55	1.53 ± 0.56	0.033
LQ_2EQL	1.10 ± 0.32	1.08 ± 0.29	1.12 ± 0.35	0.009	1.19 ± 0.43	1.18 ± 0.42	1.21 ± 0.44	0.246
LQ_3EQL	1.24 ± 0.50	1.18 ± 0.43	1.33 ± 0.56	<0.001	1.42 ± 0.60	1.42 ± 0.60	1.44 ± 0.62	0.589
LQ_4EQL	1.38 ± 0.59	1.34 ± 0.57	1.42 ± 0.61	0.021	1.67 ± 0.70	1.67 ± 0.70	1.66 ± 0.70	0.963
LQ_5EQL	1.12 ± 0.36	1.10 ± 0.32	1.15 ± 0.41	0.015	1.24 ± 0.49	1.23 ± 0.49	1.25 ± 0.48	0.339
LQ_VAS	70.51 ± 19.74	72.41 ± 18.37	68.07 ± 21.13	<0.001	64.63 ± 23.64	64.78 ± 23.2	64.08 ± 25.15	0.582
BP8	6.78 ± 1.64	6.75 ± 1.59	6.81 ± 1.70	0.119	6.35 ± 1.82	6.34 ± 1.81	6.41 ± 1.85	0.312
BP1	3.20 ± 0.75	3.18 ± 0.74	3.22 ± 0.75	0.343	2.95 ± 0.88	2.96 ± 0.88	2.93 ± 0.90	0.272

The results are presented as mean ± standard deviation.

**Table 3:** Statistics for social infrastructure features.

	Sarcopenia (%)	cul_dist	soc_dist	elder_dist	park_area	pa_area
Urban (1–8)	673/2098 (32.1%)	1232.98 ± 168.46	741.48 ± 124.86	266.00 ± 29.79	0.69 ± 0.36	8.28 ± 2.81
Rural (9–16)	550/1813 (30.3%)	1038.92 ± 97.39	764.11 ± 115.69	165.74 ± 36.31	1.25 ± 0.37	9.18 ± 3.10

The results are presented as mean ± standard deviation.

**Table 4:** Accuracy results.

Algorithms	Accuracy											
	**Basic**			**Basic + SES**			**Basic + SES + QoL**			**Basic + SES + QoL + Infra**		
	**Both**	**Male**	**Female**	**Both**	**Male**	**Female**	**Both**	**Male**	**Female**	**Both**	**Male**	**Female**
LGB	0.762	0.751	0.776	0.779	0.759	0.784	0.780	0.784	0.775	0.737	0.770	0.723
CAT	**0.769**	**0.780**	**0.788**	0.780	**0.783**	**0.796**	**0.785**	**0.790**	**0.797**	**0.749**	**0.783**	0.720
RF	0.754	0.764	0.782	**0.783**	0.780	0.791	0.779	0.786	0.790	0.733	0.762	0.726
DNN	0.738	0.738	0.747	0.741	0.740	0.750	0.734	0.749	0.747	0.721	0.737	**0.746**

Numbers in bold indicate the highest values.

**Table 5:** Precision results.

Algorithms	Precision											
	**Basic**			**Basic + SES**			**Basic + SES + QoL**			**Basic + SES + QoL + Infra**		
	**Both**	**Male**	**Female**	**Both**	**Male**	**Female**	**Both**	**Male**	**Female**	**Both**	**Male**	**Female**
LGB	0.757	0.765	0.750	0.772	0.774	0.763	0.777	0.784	0.765	0.737	0.767	**0.723**
CAT	**0.767**	**0.784**	**0.758**	0.775	**0.782**	**0.767**	**0.779**	**0.792**	**0.768**	**0.752**	**0.785**	0.717
RF	0.751	0.771	0.752	**0.776**	0.776	0.762	0.773	0.790	0.756	0.732	0.760	0.700
DNN	0.731	0.743	0.722	0.730	0.739	0.739	0.734	0.747	0.721	0.722	0.731	0.723

Numbers in bold indicate the highest values.

**Table 6:** Recall results.

Algorithms	Recall											
	**Basic**			**Basic + SES**			**Basic + SES + QoL**			**Basic + SES + QoL + Infra**		
	**Both**	**Male**	**Female**	**Both**	**Male**	**Female**	**Both**	**Male**	**Female**	**Both**	**Male**	**Female**
LGB	0.761	0.763	0.773	0.778	0.772	0.788	0.783	0.783	0.791	0.737	0.767	0.730
CAT	**0.769**	**0.782**	**0.788**	0.782	**0.781**	**0.796**	**0.785**	**0.792**	**0.797**	**0.748**	**0.783**	0.720
RF	0.754	0.770	0.776	**0.783**	0.775	0.794	0.780	0.788	0.790	0.732	0.755	0.721
DNN	0.735	0.741	0.739	0.733	0.738	0.754	0.738	0.745	0.744	0.719	0.725	**0.740**

Numbers in bold indicate the highest values.

**Table 7:** AUC results.

Algorithms	AUC											
	**Basic**			**Basic + SES**			**Basic + SES + QoL**			**Basic + SES + QoL + Infra**		
	**Both**	**Male**	**Female**	**Both**	**Male**	**Female**	**Both**	**Male**	**Female**	**Both**	**Male**	**Female**
LGB	0.814	0.837	0.750	0.821	0.840	0.757	0.824	0.856	0.750	0.754	0.838	0.653
CAT	**0.824**	**0.858**	**0.767**	**0.830**	**0.858**	**0.775**	**0.831**	**0.868**	**0.773**	**0.784**	**0.854**	**0.660**
RF	0.795	0.834	0.745	0.815	0.841	0.754	0.821	0.850	0.757	0.759	0.828	0.639
DNN	0.761	0.806	0.678	0.764	0.811	0.706	0.763	0.817	0.697	0.760	0.801	0.662

Numbers in bold indicate the highest values.

**Table 8:** AUC results with oversampling.

Algorithms	AUC with Oversampling											
	**Basic**			**Basic + SES**			**Basic + SES + QoL**			**Basic + SES + QoL + Infra**		
	**Both**	**Male**	**Female**	**Both**	**Male**	**Female**	**Both**	**Male**	**Female**	**Both**	**Male**	**Female**
LGB	0.807	0.833	0.744	0.816	0.844	0.747	0.820	0.856	0.753	0.773	0.842	0.644
CAT	**0.819**	**0.855**	**0.759**	**0.827**	**0.857**	**0.762**	**0.828**	**0.866**	**0.764**	**0.792**	**0.852**	0.672
RF	0.792	0.835	0.740	0.812	0.842	0.748	0.813	0.848	0.753	0.778	0.834	**0.679**
DNN	0.754	0.804	0.686	0.764	0.819	0.684	0.762	0.805	0.697	0.755	0.807	0.672

Numbers in bold indicate the highest values.

**Table 9:** Score of key features across prediction models.

Characteristics	LGB	CAT	RF	DNN	Score
Basic Features					
age	1	1	1	1	4
HE_BMI	1	1	1	1	4
HE_wc	1	1	1	1	4
Socioeconomic Status Features					
town_t	0	0	0	1	1
apt_t	0	0	0	1	1
incm	0	0	0	0	0
ho_incm	0	0	0	1	1
incm5	0	0	0	0	0
ho_incm5	0	0	0	0	0
ainc	1	1	1	0	3
EC1_1	0	0	0	0	0
allownc	0	0	0	0	0
house	0	0	0	0	0
live_t	1	1	0	1	3
genertn	0	0	0	0	0
cfam	0	0	0	0	0
educ	0	0	1	0	1
edu	0	0	0	0	0
marri_1	0	0	0	0	0
marri_2	1	1	1	0	3
Social Infrastructure Features					
cul_dist	1	0	0	0	1
soc_dist	1	1	1	1	4
elder_dist	1	1	1	1	4
park_area	1	1	0	0	2
pa_area	0	0	1	1	2
Quality of Life Features					
LQ_1EQL	0	0	0	0	0
LQ_2EQL	0	0	0	0	0
LQ_3EQL	0	0	0	0	0
LQ_4EQL	0	0	0	0	0
LQ_5EQL	0	0	0	0	0
LQ_VAS	0	0	1	0	1
BP8	0	0	0	0	0
BP1	0	0	0	0	0

## Data Availability

Data from the Korea National Health and Nutrition Examination Survey (KNHANES) are publicly available at [https://knhanes.kdca.go.kr/knhanes/eng/index.do] (accessed on 21 September 2023).
